# Untargeted Metabolomics Approach of Cross-Adaptation in *Salmonella Enterica* Induced by Major Compounds of Essential Oils

**DOI:** 10.3389/fmicb.2022.769110

**Published:** 2022-05-25

**Authors:** Jorge Pamplona Pagnossa, Gabriele Rocchetti, Jadson Diogo Pereira Bezerra, Gaber El-Saber Batiha, Eman A. El-Masry, Mohamed H. Mahmoud, Abdulrahman A. Alsayegh, Abdullah Mashraqi, Pier Sandro Cocconcelli, Cledir Santos, Luigi Lucini, Roberta Hilsdorf Piccoli

**Affiliations:** ^1^Health and Biological Sciences Institute, Pontifical Catholic University–PUC Minas, Poços de Caldas, Brazil; ^2^Department for Sustainable Food Process, Università Cattolica del Sacro Cuore, Piacenza, Italy; ^3^Setor de Micologia, Departamento de Biociências e Tecnologia, Instituto de Patologia Tropical e Saúde Pública, Universidade Federal de Goiás, Goiânia, Brazil; ^4^Department of Pharmacology and Therapeutics, Faculty of Veterinary Medicine, Damanhour University, Damanhour, Egypt; ^5^Microbiology and Immunology Unit, Department of Pathology, College of Medicine, Jouf University, Sakaka, Saudi Arabia; ^6^Department of Medical Microbiology and Immunology, College of Medicine, Menoufia University, Shebeen El-Kom, Egypt; ^7^Department of Biochemistry, College of Science, King Saud University, Riyadh, Saudi Arabia; ^8^Clinical Nutrition Department, Applied Medical Sciences College, Jazan University, Jazan, Saudi Arabia; ^9^Biology Department, College of Science, Jazan University, Jazan, Saudi Arabia; ^10^Department of Chemical Science and Natural Resources, Universidad de La Frontera, Temuco, Chile; ^11^Food Science Department, Federal University of Lavras, Lavras, Brazil

**Keywords:** bacterial tolerance, biofilm, terpenes, untargeted metabolomics, discriminant analysis

## Abstract

Cross-adaptation phenomena in bacterial populations, induced by sublethal doses of antibacterial solutions, are a major problem in the field of food safety. In this regard, essential oils and their major compounds appear as an effective alternative to common sanitizers in food industry environments. The present study aimed to evaluate the untargeted metabolomics perturbations of *Salmonella enterica* serovar Enteritidis that has been previously exposed to the sublethal doses of the major components of essential oils: cinnamaldehyde, citral, and linalool (CIN, CIT, and LIN, respectively). Cinnamaldehyde appeared to be the most efficient compound in the assays evaluating the inhibitory effects [0.06% (v/v) as MBC]. Also, preliminary tests exhibited a phenotype of adaptation in planktonic and sessile cells of *S*. Enteritidis when exposed to sublethal doses of linalool, resulting in tolerance to previously lethal concentrations of citral. A metabolomics approach on *S.* Enteritidis provided an important insight into the phenomenon of cross-adaptation induced by sublethal doses of major compounds of some essential oils. In addition, according to the results obtained, when single molecules were used, many pathways may be involved in bacterial tolerance, which could be different from the findings revealed in previous studies regarding the use of phytocomplex of essential oils. Orthogonal projection to latent structures (OPLS) proved to be an interesting predictive model to demonstrate the adaptation events in pathogenic bacteria because of the global engagement to prevent and control foodborne outbreaks.

## Introduction

Due to their high multiplication rates, great adaptability, and production of extracellular substances and structures, bacteria exhibit the highest microbial predominance in contaminated surfaces and equipment in the food industry (Alvarez-Ordóñez et al., 2019). Bacteria belonging to the genus *Salmonella* can form resistant and persistent biofilms on common surfaces in the food industry. In this sense, pathogenic biofilms are a potential source of contamination in food products and could explain the high number of outbreaks of foodborne diseases worldwide. Non-typhoidal *Salmonella* serovars, such as Enteritidis and Typhimurium, are etiological agents of intestinal and systemic infections, such as gastroenteritis and bacteremia, or asymptomatic chronic infections, raising awareness to the centers for epidemiological control (Piatek et al., 2019).

Several stages of food processing are vulnerable to possible sources of microbial contamination, especially in the form of biofilms. Active constituents based on acids and inorganic bases become inefficient for the sanitization process after continuous and prolonged use (Kumar and Micallef, 2017). In addition, mild concentrations of stressor agents can induce the occurrence of cellular modifications in the genetic expression, and proteomic and metabolomic profiles. The inappropriate use of antimicrobial agents in the industry has contributed to the emergence of tolerant microorganisms, which endanger the safety of food products (Cabral et al., 2016). As a result, bacterial populations develop the ability to survive a lethal exposure to common sanitizers, triggering adaptation to several types of antimicrobial molecules (Buzón-Durán et al., 2017).

The mechanisms of cross-adaptation (exposure to one stressor induces physiological and behavioral protection to a novel agent, without prior exposure to the new stressor) in complex microbial communities, such as biofilms, reduce the potential of the entire group of natural compounds (e.g., terpenes) in the prevention and control of bacterial strains (Lee et al., 2019). Therefore, it opens a gap in the prevention and control of resistant serotypes of pathogenic bacteria, such as *Salmonella* spp., making them adapted to previously effective concentrations (Buzón-Durán et al., 2017).

Nowadays, the maintenance of microbiologically safe environments in food industries is an unceasing ideal. To ensure food safety, prolong the shelf-life of food products, and satisfy consumers, a technological innovation that predicts and prevents bacterial tolerance and adaptation is required, such as machine learning and artificial intelligence (Mota et al., 2017). A search for alternatives that do not harm the health of consumers has led to the application of natural products in food production (Amuka et al., 2017).

Natural products, especially those obtained from plant extracts, act in a non-specific way in the microbial cells, without a definite target site. Vital bacterial regions, such as membrane, cellular proteins, and genetic material (DNA and RNA), are more vulnerable to the action of natural antimicrobials (Kalily et al., 2016). Besides antimicrobial effectiveness, the components of essential oils stand out positively from other antimicrobial agents, as they can reduce the emergence of tolerant pathogens, thus improving zootechnical indexes and modulating genes involved in the resistance mechanisms (Evangelista et al., 2021). Essential oils are an attractive option for natural products with antimicrobial potential. The major components of essential oils can cause structural and functional damage to the bacterial cell membrane, intracellular proteins, and enzymes (Clemente et al., 2016).

Essential oils have been used in popular medicine as an alternative treatment for a set of diseases. The effect of essential oils on the pathogenic and microbial strains causing spoilage of food has also been widely studied (Miranda et al., 2016; Tariq et al., 2019; Ghavam et al., 2020). However, considering the non-specific mode of action in cells, essential oils and/or their major compounds are still susceptible to bacterial adaptation and cross-adaptation phenomena. As previously reported in the literature, sublethal doses of natural compounds (a concentration below the required limit to inhibit or eliminate the bacterial strain) trigger physiological responses that consolidate metabolic and enzymatic apparatus to fortify the bacterial population against the next exposures (Cadena et al., 2019).

A growing interest in understanding the resistance mechanisms involved in the adaptation of bacteria in both their planktonic and biofilm state has been an important subject of research (Cadena et al., 2019). Elucidating the mechanism involved in the microbial response against chemical stressor agents is required (e.g., expulsion or consumption of active compounds). Since the over- or under-expression of cellular compounds can be decisive in bacterial survival, the presence and relative abundance of cellular metabolites are of great importance (Kongkham et al., 2020).

Metabolomics analysis, based on high-resolution mass spectrometry, is an advanced tool for the visualization of the pattern and the level of bacterial adaptation in adverse conditions. Untargeted metabolomics analysis emerges as an important tool to discover, quantify, and compare profiles of microorganisms subjected to antimicrobial agents. The functional analysis of the detected metabolites can provide a starting point for the correlation of data obtained with metabolic pathways, which provide pertinent information regarding bacterial behavior in the susceptibility tests (Cameron and Takáts, 2018). In addition, the metabolic profiles of the pathogenic bacteria can be associated with the presence and quantity of metabolites with biological roles, such as adaptive responses to sublethal doses of stressor agents (Mallick et al., 2017).

Peng et al. (2015) revised the studies of metabolomics based on a functional approach and focused on biomarker discovery and metabolome reprogramming. According to the authors, metabolomics analyses enable not only the discovery of the metabolites of cells or organisms under different conditions, but also the assessment of the frequency and relative abundance of exogenous metabolites, which have been named as discovery metabolomics. Such analyses can provide an instant overview of the physiological state of cells and indicate how the metabolic profile of a complex biological system is modified in response to stress, such as adverse factors or physiological adaptation to changes (Gibbons et al., 2015; Patel and Ahmed, 2015).

The untargeted metabolomics analysis of cells exposed to sublethal stress conditions is a semi-quantitative tool for the detection and comparison between the fundamental and stressed conditions of cells. The ultra-high-performance liquid chromatography coupled to an electron-spray ionization-quadruple time-of-flight mass spectrometry (UHPLC-ESI-QTOF-MS) is used as cutting-edge technology in untargeted metabolomics analyses. In addition, orthogonal projection to latent structures discriminant analysis (OPLS-DA), in face of the heatmap and clustering tools of the metabolomics post-tests, may establish the association between the physiological effect of sublethal exposure to natural compounds and the relative frequency and abundance of metabolites in the bacterial samples (Pagnossa et al., 2021).

Natural compounds, such as terpenes and phenylpropanoids, can be a potential source of cross-adaptation in pathogenic bacteria (Cravens et al., 2019; Álvarez-Martínez et al., 2021). Based on previous preliminary studies, cinnamaldehyde (phenylpropanoid), citral, and linalool (terpenes) represent some of the main chemical groups that show bactericidal activity among the natural compounds (de Oliveira et al., 2012; de Souza et al., 2021). Recent developments in the omics analyses have facilitated the observation of the stress responses of bacterial pathogenic strains (O’Donnell et al., 2020; Zhu et al., 2020). In this sense, a precise and comprehensive study of such a phenomenon represents a pertinent subject of investigation. This study aimed to perform an untargeted metabolomics analysis to verify the occurrence of cross-adaptation phenomena in *Salmonella enterica* serovar Enteritidis induced by the sublethal doses of major compounds of the essential oils, such as cinnamaldehyde, citral, and linalool (named as CIN, CIT, and LIN, respectively).

## Materials and Methods

### Microorganisms and Major Compounds of Essential Oils

The bacterial strains of *Salmonella enterica* serovars Enteritidis S64 and Typhimurium S190 were cordially supplied by LABENT (Laboratory of Enterobacteria) from FIOCRUZ (Oswaldo Cruz Foundation, Rio de Janeiro, Brazil). During the study, stock cultures were stored at –20°C. Subsequently, they were thawed at room temperature and reactivated by inoculating 100-μl aliquots into tubes containing 10 ml of tryptic soy broth (TSB) (Hi-Media, Mumbai, India), followed by 24 h of incubation at 37°C. The major components of essential oils, that is, cinnamaldehyde (99%, CAS: 104-55-2), citral (95%, CAS: 5392-40-5), and linalool (97%, CAS: 78-70-6), were all purchased from Sigma-Aldrich (St. Louis, Missouri, United States).

### Susceptibility Tests

The minimum bactericidal concentration (MBC) of the major components (cinnamaldehyde, citral, and linalool termed as CIN, CIT, and LIN, respectively) of essential oils were determined using a microdilution assay conducted on 96-well polystyrene plates, according to NCCLS (M7-A10) (Clinical and Laboratory Standards Institute [CLSI], 2018) with adaptations. All the TSB solutions were supplemented with 0.5% Tween 80 (an emulsifier to better homogenize the compounds present in the culture media), and each treatment including the major component of essential oils was prepared at the concentrations of 0.015, 0.03, 0.06, 0.12, 0.25, 0.50, 1.00, and 2.00% (v/v). Aliquots of 10 μl of standardized culture (2 × 10^8^ CFU ml^–1^) were added into the wells containing 190 μl of TSB medium (final inoculum at a concentration of 1 × 10^6^ CFU ml^–1^). Microplates were then incubated at 37°C for 24 h. Three aliquots of 10 μl from each well, exhibiting culture with positive growth (including concentrations twofold above and under), were then plated in Petri dishes containing tryptic soy agar (TSA) (Hi-Media, Mumbai, India) using the microdroplet technique. The MBC was determined as the lowest concentration capable of inhibiting bacterial growth. Petri dishes were incubated at 37°C for 24 h for confirmation of positive growth.

To obtain the biofilm minimum bactericidal concentration (BMBC) of major compounds, the biofilms were allowed to form in the wells of the microplates after the inoculation of aliquots (50 μl) from the standard cultures of *S.* Enteritidis and *S.* Typhimurium in 150 μl of TSB, followed by incubation at 37°C for 48 h. Cultures were then removed from the wells, and the wells were washed three times with 0.85% NaCl solution (w/v) to remove the unbounded cells. To test the biofilm susceptibility, aliquots of 200 μl of the aqueous solutions of the major components of essential oils containing 0.5% Tween 80 (v/v) were added into the wells. The concentrations used for cinnamaldehyde and citral were 0.015, 0.03, 0.06, 0.12, 0.25, 0.50, 1.00, and 2.00%, while the concentrations for linalool were 1.00, 2.00, 3.00, 4.00, 5.00, 6.00, 7.00, and 8.00%.

To quantify the obtained biofilms, the optical densities of the grown cultures were measured (600 nm) using a technique described by Merritt et al. (2005) with slight adaptations (95% ethanol was used instead of 30% acetic acid, and microplates were dried at 50°C instead of room temperature). Biofilms were classified according to Stepanović et al. (2000). The final values were the arithmetic means of the obtained absorbance readings within eight replicates.

### Adaptation and Cross-Adaptation Tests

The cultures of *S.* Enteritidis and *S.* Typhimurium were exposed to sublethal concentrations of the major essential oil components (one-fourth of the obtained MBC) (de Souza, 2016). The same concentrations of cinnamaldehyde and linalool (used for BMBC tests) were utilized, and new concentrations of citral (0.25, 0.50, 1.00, 2.00, 3.00, 4.00, 5.00, and 6.00%) were tested.

To verify the occurrence of cross-adaptation, both the *Salmonella* samples were cultured for 48 h in a TSB medium supplemented with 0.5% Tween 80 (v/v). Then, a sublethal dose of a given major component was administrated (0.06% linalool for *S.* Enteritidis, 0.03% linalool for *S.* Typhimurium, 0.015% cinnamaldehyde for both serotypes, and 0.06% citral for both serotypes) for 20 min. After this, each sample was tested against the two other major components used in the present study. For example, a 48-h culture of *Salmonella* Enteritidis was exposed to 0.015% cinnamaldehyde for 30 min and subsequently tested against citral and linalool following the susceptibility test technique.

### Untargeted Ultra-High-Performance Liquid Chromatography Coupled to an Electron-Spray Ionization-Quadruple Time-of-Flight Mass Spectrometry Profiling of *Salmonella* Enteritidis Metabolites

An aliquot of each treatment of *S.* Enteritidis standard culture (1 × 10^6^ CFU ml^–1^) was collected in amber vials for metabolomics-based profiling. Samples were analyzed in a 1290 Liquid Chromatograph coupled to a G6550 mass spectrometer through a Dual Electrospray Jet Stream ionization system (all from Agilent Technologies, Santa Clara, CA, United States). For the analyses of metabolic compounds of *S.* Enteritidis, the analytical conditions for performing UHPLC-QTOF were adopted from Rocchetti et al. (2019). According to the previously optimized analytical conditions, a reverse-phase chromatographic separation, using an acetonitrile–water binary gradient (5–90% acetonitrile in 34 min), was used. Formic acid (0.1%, v/v) was added to both acetonitrile and water, which acts as a phase modifier.

The mass spectrometer worked in full-scan mode with a nominal resolution at 30,000 FWHM and in positive polarity. For mass acquisition, a range of 100–1,200 m/z was selected. The injection volume was 6 μl considering three biological and eight analytical replicates for each sample. The injection sequence was randomized and blank samples (e.g., solvent only) were also injected. In the next step, the raw metabolomics features (e.g., mass and abundance combinations for each detected entity) were aligned and deconvoluted using the Agilent Profinder B.06 software. Data were then putatively annotated according to the ‘‘find-by-formula’’ algorithm, using the combination of monoisotopic accurate mass and the entire isotopic pattern (e.g., isotopic spacing and isotopic ratio) against MetaCyc, which is a comprehensive database on the metabolic compounds^1^.

A mass accuracy tolerance below 5 ppm was used for annotation purposes, and features were recursively identified using a retention time tolerance of ± 0.1 min for alignment (Rocchetti et al., 2018, 2019). This approach allowed gaining higher confidence in the annotation step and reaching a level 2 confidence in identification, as previously reported by the COSMOS Metabolomics Standards Initiative^2^. The Agilent Profinder B.06 software was used also used for the post-acquisition data filtering, and only those compounds putatively annotated within 100% of replications in at least one condition were retained.

### Multivariate Statistical Analyses

The Mass Profiler Professional B.12.06 (Agilent technologies) was then used for the elaboration of the untargeted UHPLC-QTOF data by unsupervised hierarchical cluster analysis (HCA), which is based on the fold-change heat map, as previously reported (Rocchetti et al., 2019). The raw metabolomics dataset was then exported and elaborated into SIMCA 13 Software (Umetrics, Malmo, Sweden) by using supervised orthogonal projections to latent structures discriminant analysis (OPLS-DA) multivariate statistics.

For the OPLS-DA model, raw data were Log2 transformed, UV scaled, and then analyzed by the OPLS-DA method. The variations between the groups were separated into predictive and orthogonal (e.g., related to technical and biological variations) components. The presence of outliers in the OPLS model was checked according to Hotelling’s T2 test (e.g., the distance from the origin in the model), using 95 and 99% confidence limits for the suspect and strong outliers, respectively.

The cross-validation (CV) of the model was then carried out using CV-ANOVA (*p* < 0.01), while permutation testing (*N* = 100) was done to exclude the overfitting of the scored data. Model parameters, for example, R^2^Y (goodness-of-fit) and Q^2^Y (goodness-of-prediction) were also recorded. Variable selection methods, such as VIP (e.g., variable importance in projection), were used to evaluate the importance of each metabolite and to select those having the highest discrimination potential (using a VIP score > 1.2 as arbitrary cut-off value). Finally, a fold-change (FC) analysis was carried out, considering the UHPLC-QTOF data, to evaluate the impact of sublethal exposure of different compounds in *S.* Enteritidis in detail.

The values obtained for the spectrophotometric measurements (mean ± standard deviation) of biofilm formation for *S*. Enteritidis and *S.* Typhimurium and each major compound (cinnamaldehyde, citral, and linalool) were subjected to ANOVA using the F-test (probability level = 1%), and Tukey’s test was performed for the significant results (probability level = 5%).

## Results and Discussion

### Susceptibility, Adaptation, and Cross-Adaptation Tests

**FIGURE 1:**
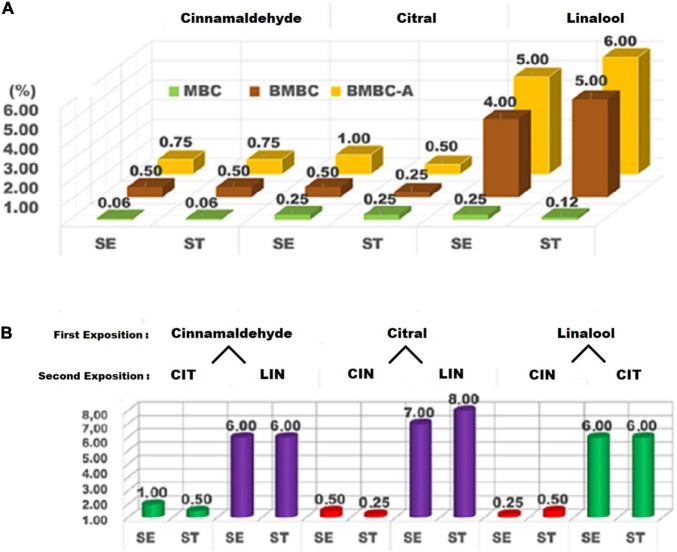
Susceptibility **(A)** and cross-adaptation tests **(B)** of *Salmonella* Enteritidis (SE) and *Salmonella* Typhimurium (ST) against cinnamaldehyde (CIN), citral (CIT), and linalool (LIN). MBC, minimum bactericidal concentration; BMBC, biofilm minimum bactericidal concentration; BMBC-A, biofilm minimum bactericidal concentration of adapted cells.

The minimum bactericidal concentration (MBC) values, MBC of biofilms (BMBC) and adapted cells (BMBC-A), and cross-adaptation tests of cinnamaldehyde, citral, and linalool are presented in Figure 1 (susceptibility tests in section “a” and cross-adaptations in section “b”). Cinnamaldehyde inhibited the growth of the planktonic cells of both *S.* Enteritidis and *S.* Typhimurium at a concentration of 0.06% (v/v). Beraldo et al. (2013) evaluated the potential of using natural products as sanitizing agents. The authors found MBCs of > 0.02% for cinnamon essential oil (67.58% cinnamaldehyde) and 0.04% for clove essential oil (77.58% eugenol) against *Salmonella* spp. in comparison to the MBC of sodium hypochlorite (0.2%), a common sanitizer in the food industry, highlighting the fact that lower doses of natural compounds were sufficient to inhibit the bacterial growth.

The bactericidal action of phenylpropanoids, such as cinnamaldehyde, and terpenes, such as citral and linalool, is still not fully understood. However, it is known that they cause the rupture of the bacterial cell membrane due to the lipophilic nature of these compounds (Chouhan et al., 2017; Tariq et al., 2019). The distortion of the physical structure of the cell and consequent membrane destabilization modify the cell permeability, causing denaturation of essential enzymes and alteration of the proton motive force. These changes result in variations in pH and electrical potential, and finally, loss of intracellular material, such as ions, ATP, and nucleic acid (Bajpai and Baek, 2016; Pisoschi et al., 2018; Nazzaro et al., 2019).

In the present study, it was also observed that the efficacy of linalool was drastically affected by the biofilm formation of *S.* Enteritidis and *S.* Typhimurium, thus requiring a concentration of up to 4.00–5.00% (v/v) to completely inhibit the bacterial growth. On the other hand, the other terpene (citral) exhibited the best performance compared to the other components (cinnamaldehyde and linalool), particularly against the sessile cells of *S.* Typhimurium. The results obtained herein corroborate the data published by Adukwu et al. (2012). In their study, the authors tested lemongrass oil containing 33% of geranial and 47% of neral (both citral isomers) against the strains of *S. aureus*, and found that the components showed high anti-biofilm activity at 0.06–0.12% concentration (v/v). The present study exposes a discrepancy in the effect exhibited by linalool (eight times less efficient than cinnamaldehyde and citral against *S.* Enteritidis biofilms, and 20 times less efficient than citral against *S.* Typhimurium).

Adaptation to the sublethal concentrations of sanitizing agents is a common event among pathogenic bacteria, as observed in *Salmonella* Typhimurium exposed to hypochlorous acid (da Cruz Nizer et al., 2020; Yuan et al., 2020); in the biofilms of *Salmonella* spp. exposed to trisodium phosphate, sodium lactate, and benzalkonium chloride; and in *Listeria monocytogenes* exposed to peracetic acid, chlorine, and quaternary ammonium salts (Alonso-Calleja et al., 2015; Márquez et al., 2018).

Zhang et al. (2014) evaluated the effects of citral, cinnamaldehyde, and tea polyphenols on the mixed biofilm formed by foodborne *Staphylococcus aureus* and *Salmonella* Enteritidis. According to the authors, anti-biofilm properties of citral and cinnamaldehyde can be attributed to a reduction in the autoinducing elements of bacterial communication signal autoinducer 2 (AI-2) in *S. aureus* and *S.* Enteritidis. Besides, Korenblum et al. (2013) evaluated the effect of citral and lemongrass oil against *Desulfovibrio alaskensis* and obtained MBC and sublethal concentrations of 0.17 mg/ml and 0.085 mg/ml, respectively. In this case, the exposure of cells (10^5^ CFU/ml) to the major components for 24 h reduced the planktonic cell population by 1 log unit and resulted in the elimination of biofilms grown on glass and steel coupons stained with iodinated propidium, whereas the control group exhibited populations of 10^7^ CFU/ml^–1^.

In the present study, to evaluate the bactericidal action of major components, susceptibility tests were performed (Figure 1A), and then cross-adaptation tests were assessed (Figure 1B). For cross-adaptation tests, the planktonic cells of *Salmonella* Enteritidis and *S.* Typhimurium were exposed to the sublethal doses of the major components of essential oils (Figure 1B). The adaptation tests of planktonic cells using one-fourth dose of the MBCs (Figure 1A) produced results similar to those obtained in the cross-adaptation tests of the BMBCs (Figure 1B).

As observed in Figure 1B, after exposure to sublethal doses of citral in *S.* Typhimurium and linalool in *S.* Enteritidis, cinnamaldehyde showed increased efficiency when compared to the previous tests (Figure 1A). To eliminate the serotypes of *Salmonella* that adapted to the sublethal doses of cinnamaldehyde and citral, increased concentrations of linalool (6.00%) were required, which demonstrates the high potential of bacterial adaptive response.

Alonso-Calleja et al. (2015) investigated the adaptability factors related to cell physiology by testing the efflux pumps and changes in the hydrophobicity of the cell surface in the strains of *E. coli.* In their experiment, cells were subjected to cross-adaptation by using common sanitizing agents in the food industry. Considering the high efficiency of different inhibitory factors when used synergistically, the authors considered that additional food safety measures should be taken when using sublethal doses.

As can also be observed in Figure 1, when *S.* Enteritidis and *S.* Typhimurium were previously exposed to sublethal doses of linalool and then tested against citral, both serotypes revealed a tolerance profile against increased concentrations (6.00%) to inhibit the biofilm formation. In other words, after a previous exposition to a low dose of linalool, *Salmonella* serotypes modified themselves to become more tolerant to a different compound (citral), thus surviving in a higher concentration of this substance. This confirmed the cross-adaptation behavior in both *Salmonella* serovars. The previous exposure to linalool may have promoted enzymatic alteration in the mechanism of bacterial resistance to terpenes, resulting in the increased tolerance to citral (de Souza, 2016). These findings suggest that the application of alternated cycles of sanitizing agents in industrial environments may not be efficient in microbiological control and safety considering the tolerance caused by sublethal doses.

According to the adopted classification (Stepanović et al., 2000), the results presented in Table 1 indicate that the unexposed cells of *S.* Enteritidis strain are strong promoters of biofilm formation, while the cells of *S.* Typhimurium exhibit a moderate capacity to develop a biofilm. In addition, a considerable increase in the mean value of measured optical densities for adapted biofilms, in comparison to those not exposed to sublethal stress (four times higher than negative control), was observed in this study (Table 2). *Salmonella* Typhimurium was classified as a strong biofilm promoter. These data confirm the increased tolerance of the *Salmonella* serotypes to major components in biofilms, which might be due to the adaptation in the planktonic phase. The increase in the biofilm formation in *Salmonella* serovars (Table 2) is affected by the exposure to natural compounds. The adaptation in the planktonic stage and biofilm formation during the sessile stage protect cells from stress and provide tolerance to new events of exposure (Singh et al., 2021).

**TABLE 1:** Spectrophotometric measurements (mean ± standard deviation) of biofilms of *S*. Enteritidis and *S.* Typhimurium.

Biofilm formation in microplate			

	Odb	Odnc	Classification
*S.* Enteritidis	0.29805 ± 0.05790a	0.05144 ± 0.00321	Strong.
*S.* Typhimurium	0.14591 ± 0.03349b	0.05246 ± 0.00648	Mod.

*Odb, optical density of biofilm; Odnc, optical density of negative control; Strong., strongly forms biofilms; Mod., moderately forms biofilms. Mean values followed by the same letter do not differ statistically by the Tukey’s test at 5% probability (a and b = between SE and ST).*

**TABLE 2:** Spectrophotometric measurements (mean ± standard deviation) of biofilm formation.

	Cinnamaldehyde			Citral			Linalool		
			
	Odb	Odcn	Class.	Odb	Odcn	Class.	Odb	Odcn	Class.
*S*E	0.5537 ± 0.1187aA	0.0503 ± 0.0005	Strong.	0.4082 ± 0.1051bA	0.0532 ± 0.0003	Strong.	0.4006 ± 0.0850bA	0.0509 ± 0.0018	Strong.
*S*T	0.1825 ± 0.0491bB	0.0564 ± 0.0018	Mod.	0.2058 ± 0.0397bB	0.0585 ± 0.0029	Mod.	0.3157 ± 0.0267aB	0.0571 ± 0.0017	Strong.

*SE, Salmonella Enteritidis; ST, Salmonella Typhimurium; Odb, optical density of biofilm; Odcn, optical density of negative control; Class., classification; Strong., strongly forms biofilm; Mod., moderately forms biofilm. Mean values followed by the same letter do not differ statistically by the Tukey’s test at 5% probability (a and b = among major compounds; A and B = between SE and ST).*

Espina et al. (2015) evaluated the effect of single sublethal doses of carvacrol, citral, and limonene on the clinical isolates of *Staphylococcus aureus* for 24 h at 37°C. After 8 h of the incubation time, a decrease in the bacterial population (1.5 log CFU ml^–1^) was observed, while the bacterial population in the negative controls of major components increased from 10^7^ to 10^8^ CFU ml^–1^. The authors stated that the incubation time should be considered as an important factor for comparison purposes between bacterial strains. In addition, according to the authors, major components continue to affect the cell viability during the planktonic phase, which may alter the biofilm formation cycle. This fact would explain the difference in cell mass observed in the presence or absence of major components.

### Untargeted Metabolomics Analysis Using Ultra-High-Performance Liquid Chromatography Coupled to an Electron-Spray Ionization-Quadruple Time-of-Flight Mass Spectrometry

In the present study, based on the results of previous studies and because of its importance in the food science scenario, the *Salmonella* Enteritidis strain S64 was used for the untargeted metabolomics analysis (de Oliveira et al., 2012; de Souza et al., 2021). The untargeted metabolomics analysis of *S.* Enteritidis treatments revealed 3,483 putatively annotated compounds (according to the level of identification as established by the COSMOS Metabolomics Initiative). In the next step, the MetaCyc database (see text footnote 1) was used in combination with the ID-Browser identification approach, provided by the Mass Profiler Professional software (Agilent), for annotation purposes. All treatments, namely, cinnamaldehyde, citral, and linalool, were submitted to a fold-change (FC) analysis, considering the pair-wise comparisons against the negative control treatment without exposure to sublethal stress concentrations (named “No stress”). This univariate analysis allowed for the observation of 276 compounds that exhibited a change of at least 1 log in relative abundance (expressed positively or negatively) when compared to the negative control.

Sublethal stress responses in *S*. Enteritidis trigger survival mechanisms aimed primarily at neutralizing harmful exogenous compounds (e.g., terpenoids) and synthesis of membrane and cell wall precursors (fatty acids and lipids). The presence of metabolites related to the degradation of natural compounds in these treatments is characterized by the non-specific response of bacteria and is related to a stress-mediated condition. This is an important point for tolerance and subsequent exposure to antimicrobial compounds (Rocha-Granados et al., 2020).

**FIGURE 2:**
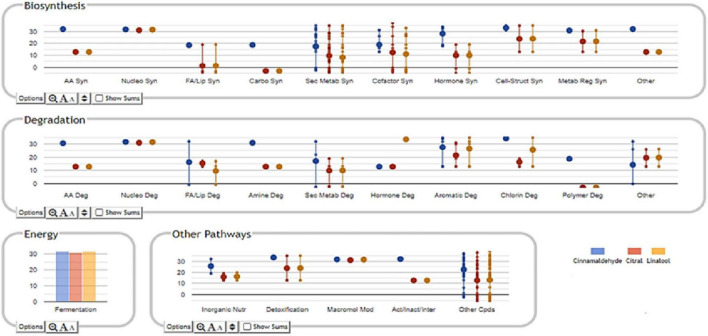
Heatmap resulting from the average hierarchical clustering considering the different *Salmonella* Enteritidis treatments. CIN, cinnamaldehyde; CIT, citral; LIN, linalool.

The unsupervised hierarchical cluster analysis (HCA) of the treatments analyzed revealed sets of distinct metabolic signatures (Figure 2). In Figure 2, it can be observed that negative control (“No Stress”) and cinnamaldehyde treatments exhibited statistically different frequencies and abundances of metabolites when compared to citral and linalool treatments. This information can be directly associated with the absence of cross-adaptation events in the bacteria exposed to sublethal stress doses of cinnamaldehyde.

**FIGURE 3:**
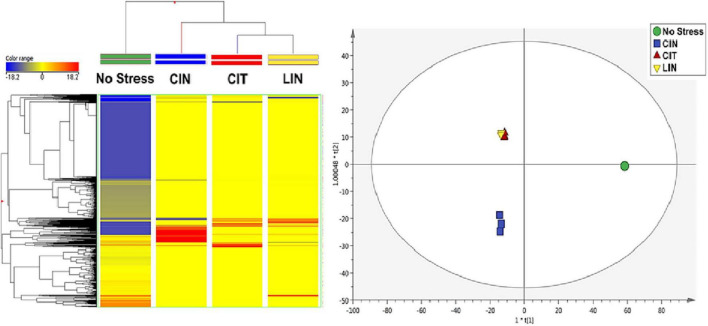
OPLS-DA of *Salmonella* Enteritidis treatments. CIN, cinnamaldehyde; CIT: citral; LIN, linalool. R^2^Y = 0.97; Q^2^Y = 0.98.

In connection with the cross-adaptation tests, based on the supervised OPLS-DA analysis (Figure 3), it was possible to observe statistical similarities in the treatments of bacteria submitted to sublethal stress concentrations of CIT and LIN. It is possible to infer that these two conditions produce an almost identical set of identified compounds, and as a consequence, a similar set of enzymes and regulatory factors are formed, leading to the sharing of very similar pathways involved in the degradation of terpenes (Rudolf et al., 2021).

**FIGURE 4:**
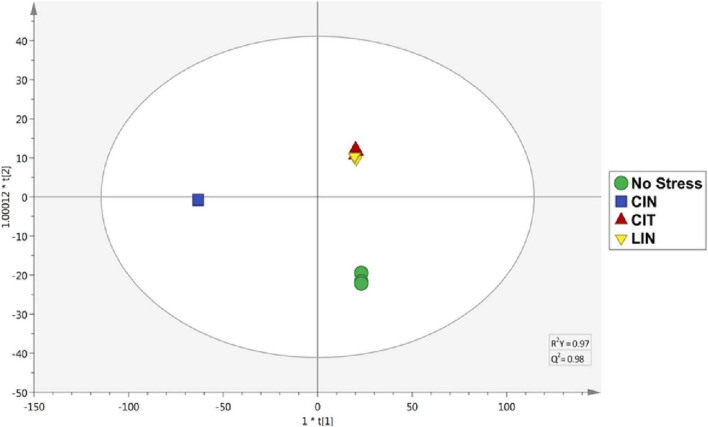
Classification of VIP markers identified in the compounds of *S.* Enteritidis by untargeted metabolomics analyses.

The selection method of VIP markers (variable importance in projection) was then used to separate the metabolites having the highest prediction score. A total of 107 metabolites were characterized by a VIP score > 1.85. These compounds have been proven to be the most discriminating metabolites and, consequently, the most critical ones involved in cell changes during sublethal stress conditions. Most of these compounds are represented by the groups of metabolites involved in secondary metabolite degradation processes, such as terpenoids, followed by biosynthesis of fatty acids and lipids (Figure 4 and Supplementary Table 1).

The Pathway Tools Omics Dashboard tool (see text footnote 1) was then used to perform a more in-depth analysis of the cellular response induced by sublethal stress concentrations of cinnamaldehyde, citral, and linalool (Figure 4). Overall, it was observed that treatment of *S.* Enteritidis with cinnamaldehyde produced higher relative frequencies and abundances of metabolites (only VIP markers) involved in biosynthesis, degradation, energy-related processes, and other pathways (inorganic nutrient metabolism and detoxification) when compared to citral and linalool treatments. It was also noticed that the metabolites identified in citral and linalool treatments occur regularly in similar relative abundances and frequencies, reinforcing the evidence for sharing analog mechanisms mediated by sublethal stress in *S.* Enteritidis (Figure 4).

Regarding the specific metabolites found in the VIP marker approach, the stress mediated by sublethal doses of cinnamaldehyde significantly increased the relative abundance of amino acids (cysteine and histidine), sterols (30-oxolanoesterol), sphingolipids (2-hydroxyhexadecanal), lipoxins (HPETE), L-arginines (y-glutamyl-L-putrescine), L-carnitines (y-butyrobetaine), and N-acetyl derivatives. The most abundant phenylpropanoid derivatives in this cluster were eugenol and similar isomers (isoeugenol and pseudoisoeugenol), which indicate a possible occurrence of cinnamaldehyde detoxification within the cell. On the other hand, the classes of metabolites that were increased following the citral and linalool treatments were those metabolites that were involved in the degradation of aromatic compounds, such as gallates, methyl gallate, vanillin, and protocatechuates. It was also observed that all treatments showed a significant increase in biosynthesis of guanine, siderophores (e.g., hydroxy-L-ornithine), folates (4-aminobenzoate), phylloquinones (dimethyl phylloquinone), nitrogenous secondary compounds (sarpagine), and D-alanyl-D-serine.

**FIGURE 5:**
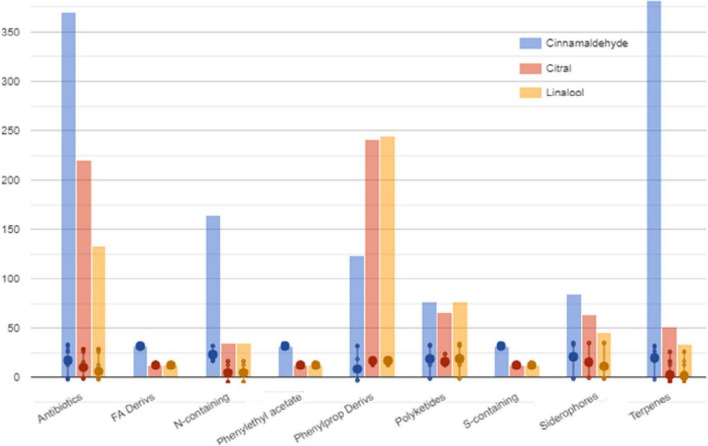
Metabolites involved in the secondary metabolite degradation during *S.* Enteritidis treatments.

Exposure to these natural compounds may interfere with the whole bacterial metabolome, thus inducing several types of biological mechanisms associated with the survival of the cell, especially related to SOS response. In addition, fatty acids and sterols play an important role in membrane and cell wall formation, resulting in the accumulation of these compounds (Pagnossa et al., 2021). An important observation regarding the terpenes involved in the degradation of secondary metabolites (Figure 5) in the negative control (no stress) is that 84% (16 out of 19) of the terpene derivatives were over-expressed in the cells treated with sublethal doses of cinnamaldehyde, which is a phenylpropanoid, while they were under-expressed in the cells treated with citral and linalool. Simultaneously, 64% (9 out of 14) of the phenylpropanoid derivatives were found in greater quantity in citral and linalool treatments, which are compounds that belong to the terpene class.

Based on these data, it is possible to consider that many of the compounds act as intermediate compounds in convergent pathways between phenylpropanoids and terpenes, resulting in the conversion of derivatives of both pathways with various metabolic functions, without necessarily culminating in the phenotypes of cross-adaptation. As proposed in previous analyses (Souza et al., 2015), adaptation tests using sublethal doses of linalool can induce a condition of tolerance in *S*. Enteritidis. Also, cross-adaptation induced by terpenes can be observed based on a high percentage of similarity in the frequencies and relative abundances of metabolites (97% of VIP markers) found in both treatments where bacteria were exposed to sublethal stress concentrations of citral and linalool.

The verification of the activation of convergent metabolic pathways can be performed through two alternatives: by metabolic flow tests using reactive carbon species or by analysis of the expression of interest genes. The RNA extraction and amplification using real-time PCR-specific gene transcripts support the correlation of data observed in metabolomics analyses with the quantification of the expression levels of interest genes (Delogu et al., 2020; Kim et al., 2020). To elucidate the participation of genes in both the metabolic pathways (degradation of phenylpropanoids and terpenes) during treatments in *S*. Enteritidis, future research is required. Finally, foodborne outbreaks worldwide can be avoided by a better understanding of the metabolic changes that occur during the sublethal exposition in food industries, thus allowing the implementation of strict measures to improve the microbiological safety of food products. Comparative studies involving the integration of OPLS-DA with machine learning, using alternative agents (e.g., nanoemulsions), can provide new insight into the techniques that can reduce the population of persistent pathogenic bacterial strains.

## Conclusion

Prudent administration and monitoring of all chemicals used in the food chain are key factors to prevent antimicrobial tolerance. Metabolomics approach on ***Salmonella*** Enteritidis provided an important insight into the finding that cross-adaptation can be induced by exposure to sublethal doses of major compounds present in some essential oils. The results obtained in the present study indicate that when single molecules are used, many pathways may be involved in inducing bacterial tolerance, and the mechanisms could be different from those revealed in the previous studies that evaluated the effect of phytocomplex of essential oils. Comprehensive discriminant analysis of OPLS has been proved to be an interesting predictive model to study the adaptation events in pathogenic bacteria, which could further facilitate the prevention and control of foodborne outbreaks across the world.

## Data Availability Statement

The original contributions presented in the study are included in the article/Supplementary Material, further inquiries can be directed to the corresponding author/s.

## Author Contributions

JP: conceptualization, methodology, investigation, writing of the original draft, and visualization. GR: data curation, investigation, formal analysis, and writing of the original draft. JB, GB, EE-M, and MM: writing, reviewing, and editing. AA and AM: data analyses and revision of the manuscript. PC: methodology and investigation. CS: project conceptualization and guidance, manuscript writing, and revision. LL: supervision, methodology, validation, resources, and funding acquisition. RH: conceptualization, project administration, supervision, methodology, validation, resources, and funding acquisition. All authors contributed to the article and approved the submitted version.

## Conflict of Interest

The authors declare that the research was conducted in the absence of any commercial or financial relationships that could be construed as a potential conflict of interest.

## Publisher’s Note

All claims expressed in this article are solely those of the authors and do not necessarily represent those of their affiliated organizations, or those of the publisher, the editors and the reviewers. Any product that may be evaluated in this article, or claim that may be made by its manufacturer, is not guaranteed or endorsed by the publisher.

## Footnotes

1
http://metacyc.org

2
http://cosmos-fp7.eu/msi
