# Supplementary material for: Untargeted Metabolomics Approach of Cross-Adaptation in Salmonella Enterica Induced by Major Compounds of Essential Oils
Source: Front Microbiol. 2022 May 25;13:769110. doi: 10.3389/fmicb.2022.769110 (PMC9174793; doi:10.3389/fmicb.2022.769110)
Supplement: Supplementary file 1 [file Table_1.pdf]

Supplementary Table 1

Var ID (Primary)	M1.VIP[3+2+0]	2.44693 * M1.VIP[3]cvSE
lividomycin B	2,7601	0,796074
synechoxanthin	2,7506	0,793319
dihydrostreptomycin	2,7497	0,79299
3-hydroxy-4-methyl-anthranilate pentapeptide lactone	2,7486	0,793067
atabrine	2,7477	0,792083
6'''-deamino-6'''-hydroxyneomycin C	2,7419	0,789537
paromomycin	2,7419	0,789537
5-iO/i-&beta\-mycaminosyltylonolide	2,7419	0,789537
sucrose-3-decanoyl-4-isoveleroyl-3'-isovalerate	2,7388	0,789042
simvastatin lactone	2,737	0,789716
desferrioxamine E	2,7366	0,789895
15-oxo spinosyn macrolactone	2,735	0,785635
huvastatin lactone	2,735	0,785635
sucrose-3-(2-methyl)butyryl-4-isovaleroyl-3'-laurate	2,7339	0,791532
sucrose-3-lauroyl-4-isovaleroyl-3'-isovalerate	2,7339	0,791532
sucrose 3-lauroyl-4-isovaleroyl-3'-(2-methyl)butyrate	2,7339	0,791532
iCypridina/i oxyluciferin	2,7295	0,785231
phytosphingosine 1-phosphate	2,7295	0,785269
prefusarin (open ring form)	2,7295	0,784695
narbonolide	2,7084	0,775984
2-palmitoyl-trehalose	2,5357	0,791206
terfenadine	2,521	0,742374
apicidin D2	2,5134	0,755217
(2S*3R*4S)-4-hydroxy-L-isoleucine	2,4928	0,680832
azaguanine	2,4928	0,680832
&beta\-guinidoxypropionamide	2,4928	0,680832
5-hydroxy-leucine	2,4928	0,680832
4-hydroxy-L-leucine	2,4928	0,680832
sucrose 2-isovaleroyl-3-isodecanoyl-4-isobutanoate	2,461	0,770946
sucrose 3-lauroyl-4-isovaleroyl-3'-acetate	2,461	0,770946
fusidate	2,428	0,75157
2-stearoyl-trehalose	2,4246	0,722575
&alpha\-paxitriol	2,4244	0,67134
&beta\-paxitriol	2,4244	0,67134
digitoxigenin 3-O-&beta\-D-quinovoside	2,4145	0,81876
digiproside	2,4145	0,81876
terpendole C	2,4115	0,81786
sucrose 3-lauroyl-3'-isovalerate	2,4013	0,718028
sucrose-3-lauroyl-4-isovalerate	2,4013	0,718028
2'*3'*4'-iO/i-methyl-rhamnosyl tetracyclic spinosyn	2,4003	0,716858
20-hydroxy-5-iO/i-mycaminosyltylactone	2,3988	0,716238
&beta\-aflatrem	2,3785	0,806443
aflatrem	2,3785	0,806443
guadinomine B	2,3784	0,806418
westiellamide	2,3706	0,751907
2'-iO/i-methyl-rhamnosyl tetracyclic spinosyn	2,3663	0,749702
1-(&alpha\-linolenoyl)-isn/i-glycero-3-phosphoethanolamine	2,3543	0,743396

methymycin	2,3543	0,743395
neomethymycin	2,3543	0,743395
ethyl-2-methylacetoacetate	2,3006	0,621887
pentachlorophenol	2,2491	0,761667
13-desoxypaxilline	2,1191	0,587208
1-acyl-sn-glycero-3-phosphoglycerol (n-C16:0)	2,0915	2,52297
2-16:0-lysophosphatidylglycerol	2,0915	2,52297
7*8-diketopelargonate	2,0495	0,497965
6-isobutyl-4-hydroxy-2-pyrone	2,0495	0,497965
veratryl alcohol radical	2,0495	0,497965
3-[(1iS/i)-cyclohex-3-en-1-yl)]-2-oxopropanoate	2,0495	0,497965
ubiquinol-0	2,0481	0,49722
veratraldehyde	2,0481	0,49722
3-methylorcinaldehyde	2,0481	0,49722
3-methoxy-4-hydroxyphenylglycol	2,0481	0,49722
L-mycarose	2,0481	0,49722
1-(2*4-dihydroxy-3-methylphenyl)ethanone	2,0481	0,49722
(iR/i)-1*3-diphenyl-pentane-1*4-dione	2,0155	1,6497
(7R)-itrans/i-hinokiresinol	2,0155	1,6497
(7S)-itrans/i-hinokiresinol	2,0155	1,6497
L-alanyl-D-glutamate	1,954	1,49227
&gamma\-L-glutamyl-D-alanine	1,954	1,49227
20-dihydrorosamicin	1,9259	0,551681
(3R)-3-hydroxy-16-methoxy-2*3-dihydrotabersonine	1,9171	1,57089
deacetylvindorosine	1,9171	1,57089
sphinganine 1-phosphate	1,9154	1,57001
16-methoxytabersonine	1,9148	1,56856
11-dehydro-15-oxo spinosyn macrolactone	1,9133	1,56605
spinosyn tricyclic macrolactone	1,9133	1,56605
12-&alpha\-hydroxy-3 oxochola-4*6-dienoate	1,9128	1,5624
7-&alpha*12-&alpha\-dihydroxy-3-oxochol-4-enoate	1,9128	1,5624
3 &beta\-hydroxy-7*12-diketocholanate	1,9128	1,5624
10&beta*14&beta\-dihydroxytaxa-4(20)*11-dien-5&alpha\-	1,9091	1,55805
1-oleoyl-isn/i-glycero-3-phospho-(1'-isn/i-glycerol)	1,888	1,52892
xylostasin	1,884	0,562143
(behenoyl)adenylate	1,864	0,533323
furostanol-26-alcohol	1,858	0,707223
2*22-dideoxyecdysone	1,858	0,707223
calcitetrol	1,858	0,707223
glycolithocholate	1,858	0,707223
(25iR/i)-3&alpha*7&alpha*12&alpha\-trihydroxy-5&beta\-	1,8574	0,707409
4*4'-diaponeurosporenoate	1,8574	0,707408
7'-O-demethylcephaeline	1,8573	0,707772
solasodine	1,8572	0,708391
piericidin A	1,8569	0,707024
&beta\-tocotrienol	1,8569	0,707024
&gamma\-tocotrienol	1,8569	0,707024
2*3-dimethyl-6-geranylgeranyl-1*4-benzoquinol	1,8569	0,707024
4*4'-diapolycopene-4-al	1,8563	0,706824
3*7*12-trihydroxycoprostanate	1,8552	0,708082

**lyngbyatoxin C**	1,855	0,706854
**huvastatin acid**	1,855	0,706849
**(i22R*23R/i)-28-homocastasterone**	1,8548	0,707062
**lavendomycin**	1,8517	0,538731
**3-[(3Z*6Z*9Z*12Z)-pentadeca-3*6*9*12-tetraen-1-yl]-4-[(2Z*5Z*8Z*11Z)-tetradeca-2*5*8*11-tetraen-1-yl]oxetan-2-**	1,8514	0,707446
**oleanolic aldehyde**	1,8508	0,707101
**(4Z*8Z*12Z*16Z*20Z)-4*8*12*16*20-pentamethyl-24-**	1,8508	0,707101
**11-oxocucurbitadienol**	1,8508	0,707101
**4&alpha\-formyl*4&beta*14&alpha\-dimethyl-9&beta*19-cyclo-5&alpha\-cholest-24-en-3&beta\-ol**	1,8508	0,707101
