# Supplementary figures and images for: Untargeted Metabolomics Approach of Cross-Adaptation in Salmonella Enterica Induced by Major Compounds of Essential Oils
Source: Front Microbiol. 2022 May 25;13:769110. doi: 10.3389/fmicb.2022.769110 (PMC9174793; doi:10.3389/fmicb.2022.769110)

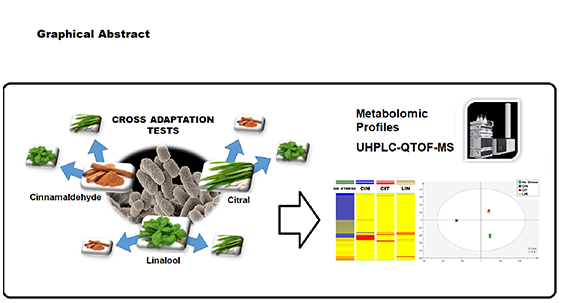

Supplement: Supplementary file 2 [file Image_1.tif]
